# Accurate prediction of biliary atresia with an integrated model using MMP-7 levels and bile acids

**DOI:** 10.1007/s12519-023-00779-7

**Published:** 2023-12-23

**Authors:** Yi-Jiang Han, Shu-Qi Hu, Jin-Hang Zhu, Xiao Cai, Deng-Ming Lai, Bao-Hai Chen, Kun Zhu, Qiao Tong, Xin-Rui Zhou, Jia-Le Deng, Jin-Fa Tou, Zhuo Fang, Li-Zhong Du

**Affiliations:** 1 Department of Neonatal Surgery, Children’s Hospital, Zhejiang University School of Medicine, National Clinical Research Center for Child Health, Hangzhou, China; 2 Department of Data and Analytics, WuXi Diagnostics Innovation Research Institute, Shanghai, China; 3 Department of Information Center, Children’s Hospital, Zhejiang University School of Medicine, National Clinical Research Center for Child Health, Hangzhou, China; 4 Department of Pathology, Children’s Hospital, Zhejiang University School of Medicine, National Clinical Research Center for Child Health, Hangzhou, China; 5 Department of Neonatology, Children’s Hospital, Zhejiang University School of Medicine, National Clinical Research Center for Child Health, Hangzhou, China

**Keywords:** Bile aid, Biliary atresia, Diagnosis, Liver fibrosis, Liver test, MMP-7

## Abstract

**Background:**

Biliary atresia (BA) is a rare fatal liver disease in children, and the aim of this study was to develop a method to diagnose BA early.

**Methods:**

We determined serum levels of matrix metalloproteinase-7 (MMP-7), the results of 13 liver tests, and the levels of 20 bile acids, and integrated computational models were constructed to diagnose BA.

**Results:**

Our findings demonstrated that MMP-7 expression levels, as well as the results of four liver tests and levels of ten bile acids, were significantly different between 86 BA and 59 non-BA patients (*P* < 0.05). The computational prediction model revealed that MMP-7 levels alone had a higher predictive accuracy [area under the receiver operating characteristic curve (AUC) = 0.966, 95% confidence interval (CI): 0.942, 0.989] than liver test results and bile acid levels. The AUC was 0.890 (95% CI 0.837, 0.943) for liver test results and 0.825 (95% CI 0.758, 0.892) for bile acid levels. Furthermore, bile levels had a higher contribution to enhancing the predictive accuracy of MMP-7 levels (AUC = 0.976, 95% CI 0.953, 1.000) than liver test results. The AUC was 0.983 (95% CI 0.962, 1.000) for MMP-7 levels combined with liver test results and bile acid levels. In addition, we found that MMP-7 levels were highly correlated with gamma-glutamyl transferase levels and the liver fibrosis score.

**Conclusion:**

The innovative integrated models based on a large number of indicators provide a noninvasive and cost-effective approach for accurately diagnosing BA in children.

Video Abstract (MP4 142103 KB)

**Supplementary Information:**

The online version contains supplementary material available at 10.1007/s12519-023-00779-7.

## Introduction

Biliary atresia (BA) is a rare progressive hepatic disease in children with unclear etiology [1, 2]. It is caused by progressive inflammation and fibrosis, which interrupts bile flow and eventually causes severe hepatic injury [3]. The incidence of BA is 0.7-3.2 per 10,000 live births in Asia, which is higher than that in Western countries [4–6]. Without effective treatment, progressive liver cirrhosis will lead to death in BA patients. Currently, the effective treatment strategy for BA patients is Kasai portoenterostomy (KPE) followed by liver transplantation if necessary. Early treatment using KPE will increase the survival time and decrease the need for liver transplant; however, the KPE treatment window is narrow [7–9]. The reference standard diagnostic method for BA is intraoperative cholangiography, which is highly invasive. Many children with BA have end-stage liver cirrhosis because of delayed diagnosis. Therefore, timely and noninvasive diagnostic approaches are urgently needed [10, 11].

The serum MMP-7 level is significantly higher in infants with BA than in infants without BA [12]. Some studies have hypothesized that MMP-7 expression is correlated with BA, while different studies have reached contrary conclusions [12, 13]. A large-scale proteomics study has revealed that MMP-7 is an indicator of BA, and the diagnostic accuracy is very high [13]. MMP-7 is supposed to be the next best serum biomarker for BA; however, the MMP-7 cutoff values for BA diagnosis vary among studies [14].

Bile acids are synthesized by liver cells, and dysregulated bile acid synthesis and metabolism often induce abnormal liver function [15, 16]. Serum bile acid levels are sensitive indicators of hepatic disease and are useful to determine liver injury [17, 18]. In addition, serum liver tests are used to determine liver injury. Several studies reported that some of these plasma elements could distinguish BA and non-BA patients [19]. Gamma-glutamyl transferase (GGT) and aspartate aminotransferase (ALT) levels were reported to have high accuracy and specificity for the diagnosis of BA [20]. The ratio of aspartate aminotransferase (AST) to blood platelets was the clinical marker for BA diagnosis, and a prospective study revealed that bile acid combined with GGT levels had a high diagnostic accuracy for estimating the risk of BA in infants with cholestasis [21]. Cholestasis in children is typically defined as a condition involving a problem with bile formation or flow within the first year of life, often occurring in the first three months. This condition leads to the buildup of bile and related substances within the liver, which can cause severe damage to the liver [22]. However, there is a lack of integrated algorithms to predict BA using MMP-7 levels combined with bile acid levels and liver test results.

In this study, our primary objective was to develop an accurate diagnostic method for BA. To achieve this goal, we employed a comprehensive approach. We compared serum MMP-7 levels, liver test results, and bile acid levels between a BA group and a non-BA group. Additionally, we explored the correlation between serum MMP-7 levels and the degree of fibrosis. Furthermore, we developed computational models using MMP-7 levels alone, liver test results, and bile acid levels. Finally, we created integrated models that combine MMP-7 levels with either liver test results or bile acid levels. These innovative integrated models offer a noninvasive and cost-effective approach for the accurate diagnosis of biliary atresia in children.

## Methods

### Study participants

Infants with cholestasis aged less than five months were consecutively enrolled in an observational study between April 2021 and February 2023 at Children’s Hospital, Zhejiang University School of Medicine, National Clinical Research Center for Child Health, Hangzhou, China. The inclusion criteria for infants with cholestasis were as follows: (1) a serum direct bilirubin level > 17.1 µmol/L when the total bilirubin level was < 85.5 µmol/L or (2) a direct component > 20% of the total when the total bilirubin level was > 85.5 µmol/L. Blood samples were collected when the children were diagnosed with cholestasis. The diagnosis of BA was made through intraoperative cholangiography and histological examination of liver biopsies, and the pathologist was blinded to whether the infant had been diagnosed with BA. The exclusion criteria of BA patients were as follows: (1) cholestasis patients with intact biliary trees on cholangiography, (2) genetic analysis conforming to the etiology. Written informed consent was obtained from all the children’s caregivers. This study was approved by the Ethics Committee of Children’s Hospital, Zhejiang University School of Medicine (2023-IRB-0066-P-01).

### Serum MMP-7 level measurement

Serum MMP-7 levels were measured using an enzyme-linked immunosorbent assay (ELISA) kit (R&D Systems, DMP700, Minneapolis, MN, USA) according to the manufacturer’s protocol. All measurements were performed by WuXi Diagnostics (Shanghai, China), who were blinded to the other test results and final diagnosis. Serum samples were diluted 20 times. All samples were assayed in triplicate, and the mean serum MMP-7 level was analyzed. Only 5 hours were needed for MMP-7 measurement using the enzyme-linked immunosorbent assay (ELISA) kit, which is a clinically available test.

### Bile acid profile measurement

Serum samples were collected from all 145 participants and stored at − 80 °C. After complete melting at room temperature, 20 μL of internal standard solution was added to 60 μL of serum sample and mixed for 0.5 minute in a 1.5-mL Eppendorf tube. Then, 240 μL of acetonitrile was added to the mixture and mixed for 1 minute. The tube was then centrifuged at 12,000 rpm for 5 minutes. Subsequently, 80 μL of supernatant was extracted into a 96-well plate containing 160 μL of pure water. The plate was then vibrated for 5 minutes in the oscillator and centrifuged for 5 minutes at 3000 rpm. Finally, 2 µL of the resulting solution was injected into a liquid chromatography-tandem mass spectrometry (LC-MS/MS) (Xevo TQ-S, Waters, USA) system for measurement.

### Liver tests

For each infant, 3 ml of venous blood was collected in a tube containing inert separation gel and procoagulant, which was centrifuged for 10 minutes and used for the detection of liver function items. The instrument was a Beckman Coulter automatic biochemical analyzer (AU5800, USA). Total protein (TP) levels were measured using the biuret method, albumin (ALB) levels were assessed using the bromocresol green method, and globulin (GLO) levels, the albumin/globulin ratio (A/G), and indirect bilirubin (IB) levels were assessed using the calculation method. Total bilirubin (TB) levels and direct bilirubin (DB) levels were determined using the dichlorphenyldiazonium (DPD) method. The levels of AST, ALT, cholinesterase (CHE), alkaline phosphatase (ALP), adenosine deaminase (ADA), lactate dehydrogenase (LDH) and GGT were detected by the rate method. Prealbumin (PAB) levels were determined using the turbidimetric immunoassay method, and cholesterol (CHOL) levels were assessed using an enzymatic method.

### Liver fibrosis scores

The degree of liver fibrosis in the 106 participants, including 84 BA patients and 22 non-BA patients, was classified using the Ohkuma classification method. Among the 22 non-BA patients, laparoscopic biliary duct exploration, cholangiography, and liver tissue biopsy were performed to rule out BA. The Ohkuma classification of liver fibrosis is typically performed using liver tissue obtained from a liver biopsy. Liver specimens were prepared for examination, which involved fixing the tissue in formalin and embedding it in paraffin wax. The tissue was then sliced into thin sections and stained using H&E staining. The sections were then examined under a microscope, and the degree of fibrosis was assessed based on the severity of collagen deposition. The degree of liver fibrosis was scored according to the Ohkuma classification as follows: Grade 0 indicated no fibrosis; Grade 1 indicated fibrosis that was confined to the portal area; Grade 2 indicated mild bridging fibrosis extending to the neighboring portal area; Grade 3 indicated widened bridging fibrosis, and Grade 4 indicated pseudolobule formation [23]. An experienced pathologist determined the appropriate stage based on the degree of fibrosis observed in the liver tissue.

### Sample size and power calculation

In the current study, a power calculation was performed to ensure the adequacy of our sample size for discerning the utility of MMP-7 levels in distinguishing between BA and non-BA patients. The pwr.t2n.test function from the pwr package in R was used to compute the power of the test or determine parameters to obtain a target power for a two-sample *t* test with different sample sizes. Utilizing the observed difference in means of 56.7 and a pooled standard deviation of 43.3 with an alpha set at 0.05 and considering our sample sizes of 86 patients in the BA group and 59 patients in the non-BA group, our analysis yielded a power value of 1. This indicated near certainty for detecting a true difference between the groups, given that one existed. Such a high power further highlights the robustness of our sample size, underscoring its sufficiency in supporting the primary objective of our investigation.

### Statistical analysis

The demographic and clinical characteristics of the patients are summarized using conventional descriptive statistics: *n* (%) for categorical variables and medians and quartiles (25% quantile, 75% quantile) for continuous variables. Between-group comparisons were performed using the chi-square and Mann-Whitney *U* tests. A statistically significant difference was defined as a *P* value < 0.05. Boxplots were created to observe differences in biomarkers between the BA group and the non-BA group. *P* values in the boxplots were calculated using Mann-Whitney *U* tests. For variable selection, least absolute shrinkage and selection operator (LASSO) models were initially applied to handle multicollinearity, which occurs when two or more predictor variables are highly correlated. Stepwise regression was then applied based on the Akaike information criterion (AIC). The model with the lowest AIC was selected as the model with the best tradeoff between fit and model complexity. The variables in that specific model were determined as the final set of selected variables. Logistic regression was applied to construct all models using the selected variables. Receiver operating characteristic (ROC) curves were constructed, and the area under the curve (AUC) and 95% confidence intervals (CIs) are reported as a measure of model performance. The optimal cutoffs were defined based on achieving the largest specificity for maximum sensitivity. The sensitivity, specificity, positive predictive value (PPV), and negative predictive value (NPV) were also used to show diagnostic accuracy. The AST-to-platelet ratio index (APRI) formula is as follows [24]: APRI = (AST/upper limit of normal for AST) × 100/platelet count (10^9^/L). In this formula, AST represents the concentration of aspartate aminotransferase in the serum, and platelet count refers to the number of platelets in the blood. The upper limit of normal for AST refers to the upper limit of the normal range for the aspartate aminotransferase concentration. All data analyses were performed using R software 4.2.0 (R Foundation, Vienna, Austria).

## Results

### Clinical characteristics of participants

The study included 145 individuals, including 86 BA patients and 59 non-BA patients. The median age of the patients was 54 days, and their ages ranged from 2 to 148 days. Their demographic information including sex, age, gestational week, birth weight, age at surgery, degree of hepatic fibrosis, and BA type is shown in Table 1. The final diagnoses of cholestasis in infants without BA (*n* = 59) included Alagille syndrome (*n* = 2), intrahepatic bile duct dysplasia (*n* = 8), congenital choledochal cyst (*n* = 1), Lucey-Driscoll syndrome (*n* = 1), progressive familial intrahepatic cholestasis (PFIC) (*n* = 2), congenital hypothyroidism (*n* = 2), parenteral nutrition–associated cholestasis (PNAC) (*n* = 14), cytomegalovirus (CMV) hepatitis (*n* = 3), Rotor syndrome (*n* = 1), citrin deficiency (*n* = 2) and idiopathic cholestasis (*n* = 23). The degree of liver fibrosis was scored according to the Ohkuma classification, resulting in a score ranging from 0 to 4 for each participant. The results showed that there were significant differences in sex, gestational week, birth weight, and fibrosis score between the BA and non-BA groups (*P* < 0.05).

**Table 1:** Participants demographics

Variables	Overall	Biliary atresia (BA)	Non-BA	*P*-value^a^
Diagnosis status	145	86	59	
Sex (male %)	84 (58%)	39 (45%)	45 (76%)	< 0.001
Age (d)	54 (33, 72)	51 (24, 64)	56 (44, 84)	0.021
Gestational week	38 (36, 38)	38 (38, 39)	36 (31, 38)	< 0.001
Birth weight (kg)	2.92 (2.36, 3.26)	3.10 (2.81, 3.40)	2.35 (1.45, 3.00)	< 0.001
Surgery age (d)	57 (35, 72)	55 (30, 67)	60 (48, 84)	0.080
Ohkuma (*n*, %)	106	84	22	< 0.001
0	14 (13%)	3 (3.6%)	11 (50%)	
1	27 (25%)	18 (21%)	9 (41%)	
2	31 (29%)	29 (35%)	2 (9.1%)	
3	20 (19%)	20 (24%)	0 (0%)	
4	14 (13%)	14 (17%)	0 (0%)	
BA type (*n*, %)	86	86	0	> 0.9
I	3 (3.5%)	3 (3.5%)	0 (0%)	
IIa/b	0 (0%)	0 (0%)	0 (0%)	
III	83 (96.5%)	83 (96.5%)	0 (0%)	

Continuous variables are shown in median interquartile range

^a^Pearson’s Chi-squared test for sex; Wilcoxon rank sum test for age; Fisher's exact test for Ohkuma

### Significantly different indicators between biliary atresia and non-biliary atresia patients

**Fig. 1:**
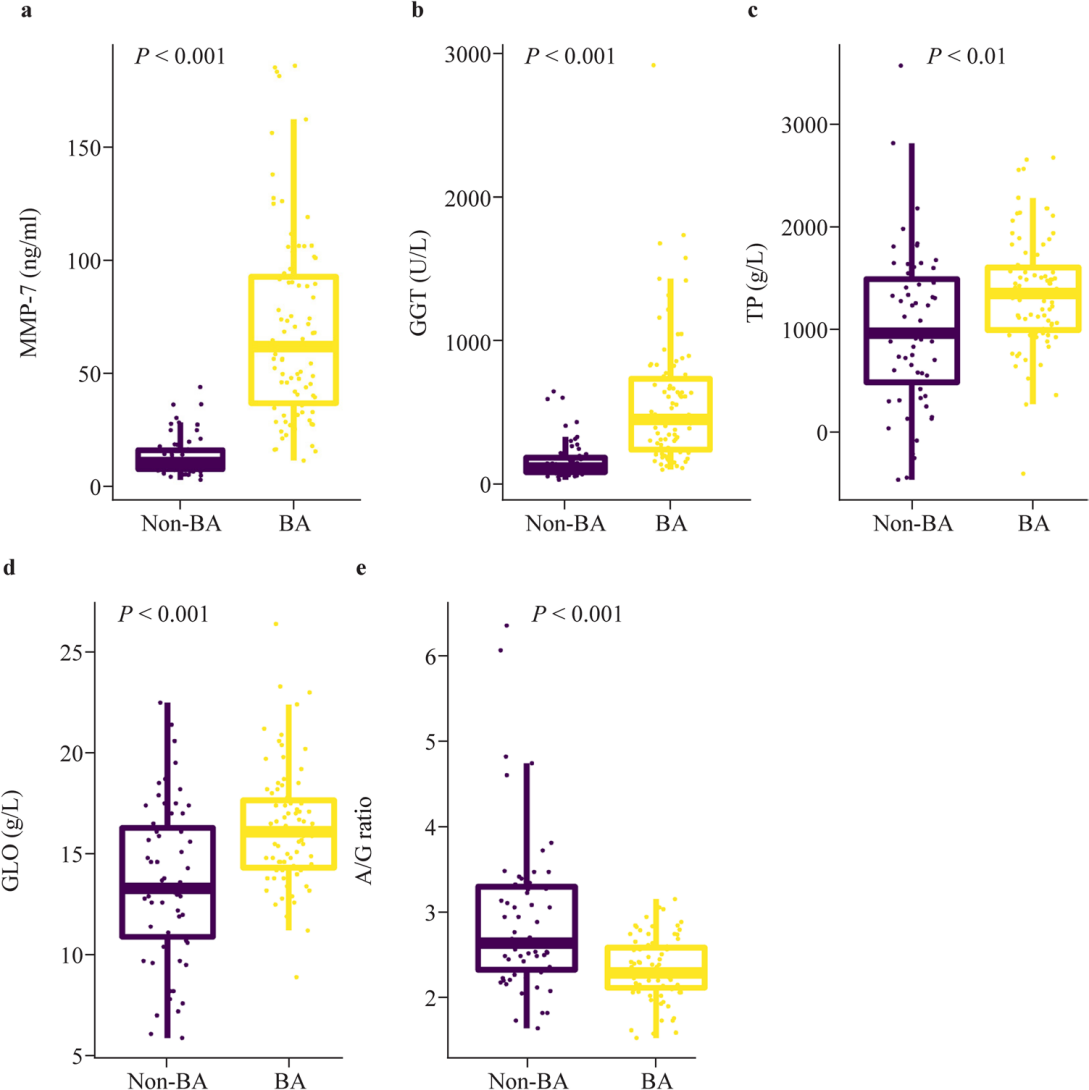
Significantly different levels of MMP-7 and liver tests between the BA and non-BA patients. **a**–**e** Represent the levels comparison of MMP-7, GGT, TP, GLO, and A/G ratio between the BA and non-BA groups. The BA group comprised 86 patients, and the non-BA group comprised 59 patients. Chi-squared and Mann-Whitney *U* tests were used for between-group comparisons, and a *P* value of less than 0.05 was considered statistically significant. *MMP-7* Matrix metalloproteinase-7, *BA* biliary atresia, *GGT* gamma-glutamyl transferase, *TP* total protein, *GLO* globulin, *A/G ratio* albumin to globulin (A/G) ratio

**Fig. 2:**
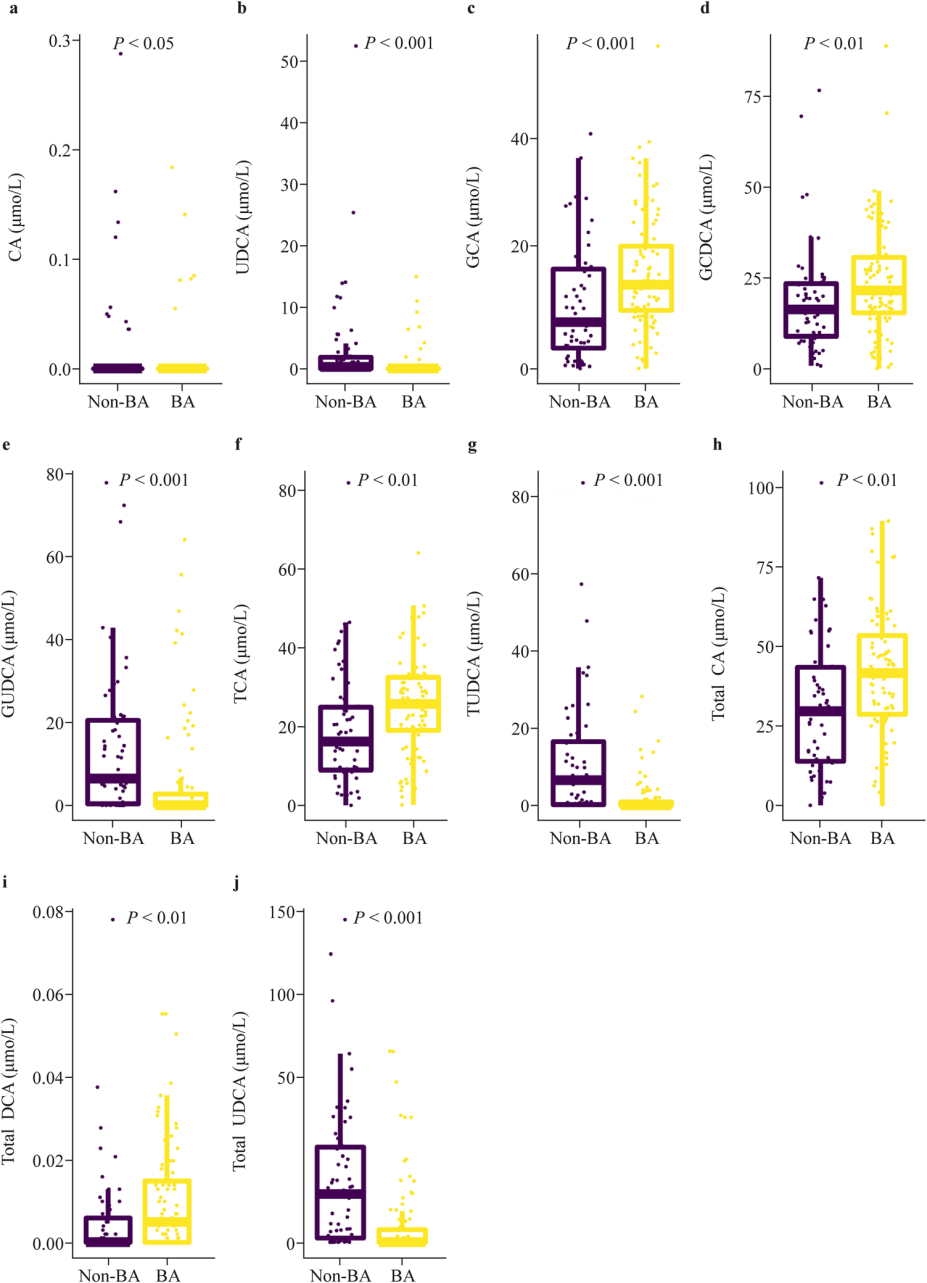
Significantly different indicators of bile acid levels between BA and non-BA participants. **a**–**j** Represent the level comparisons of CA, UDCA, GCA, GCDCA, GUDCA, TCA, TUDCA, total CA, Total DCA, and total UDCA between the BA and non-BA groups. The BA group comprised 86 patients, and the non-BA group comprised 59 patients. Chi-squared and Mann-Whitney *U* tests were used for between-group comparisons, and a *P* value of less than 0.05 was considered statistically significant. *BA* biliary atresia, *CA* cholic acid, *UDCA* ursodeoxycholic acid, *GCA* glycocholic acid, *GCDCA* glycochenodeoxycholic acid, *GUDCA* glycoursodeoxycholic acid, *TCA* taurocholic acid, *TUDCA* tauroursodeoxycholic acid, *Total CA* CA + TCA + GCA, *DCA* deoxycholic acid, *TDCA* taurodeoxychoic acid, *GDCA* glycodeoxycholic acid, *Total DCA* DCA + TDCA + GDCA, *TCDCA* taurochenodeoxycholic acid, *Total UDCA* UDCA + TUDCA + GUDCA

MMP-7 expression levels, the results of 13 liver tests (Supplementary Table 1), and the levels of 20 bile acids were determined for all BA and non-BA patients (Supplementary Table 2). The median serum MMP-7 level for the BA patients was 61.95 ng/mL, with an interquartile range of 36.94–92.94 ng/mL. The median serum MMP-7 level for the non-BA patients was 10.79 ng/mL, with an interquartile range of 8.02–16.25 ng/mL. The MMP-7 expression level was significantly higher in BA patients than in non-BA patients (Fig. 1a**)**. For the liver tests, the GGT, TP, and GLO levels were significantly higher in BA patients than in non-BA patients (Fig. 1b–d), while the A/G ratio was significantly lower in BA patients than in non-BA patients (Fig. 1e). Detailed information on the comparison results of MMP-7, GGT, TP, and GLO levels and the A/G ratio is shown in Supplementary Table 1. For bile acid levels, glycocholic acid (GCA), glycochenodeoxycholic acid (GCDCA), taurocholic acid (TCA), total CA [cholic acid (CA) + GCA + TCA], and total DCA [deoxycholic acid (DCA) + taurodeoxycholic acid (TDCA) + glycodeoxycholic acid (GDCA)] levels were higher in BA patients than in non-BA patients (Fig. 2c, d, f, h, i), while CA, ursodeoxycholic acid (UDCA), glycoursodeoxycholic acid (GUDCA), tauroursodeoxycholic acid (TUDCA), and total UDCA (UDCA + TUDCA + GUDCA) levels were significantly lower in BA patients than in non-BA patients (Fig. 2a, b, e, g, j). Detailed information on the comparison results of the levels of these bile acids is shown in Supplementary Table 2. In addition, we compared the expression levels of MMP-7 in BA and non-BA patients whose ages were under 30 days and under 10 days, and the MMP-7 expression level was also significantly different between BA and non-BA patients (Supplementary Fig. 1).

### MMP-7 alone had a higher predictive ability for biliary atresia than liver test results and bile acid levels

**Fig. 3:**
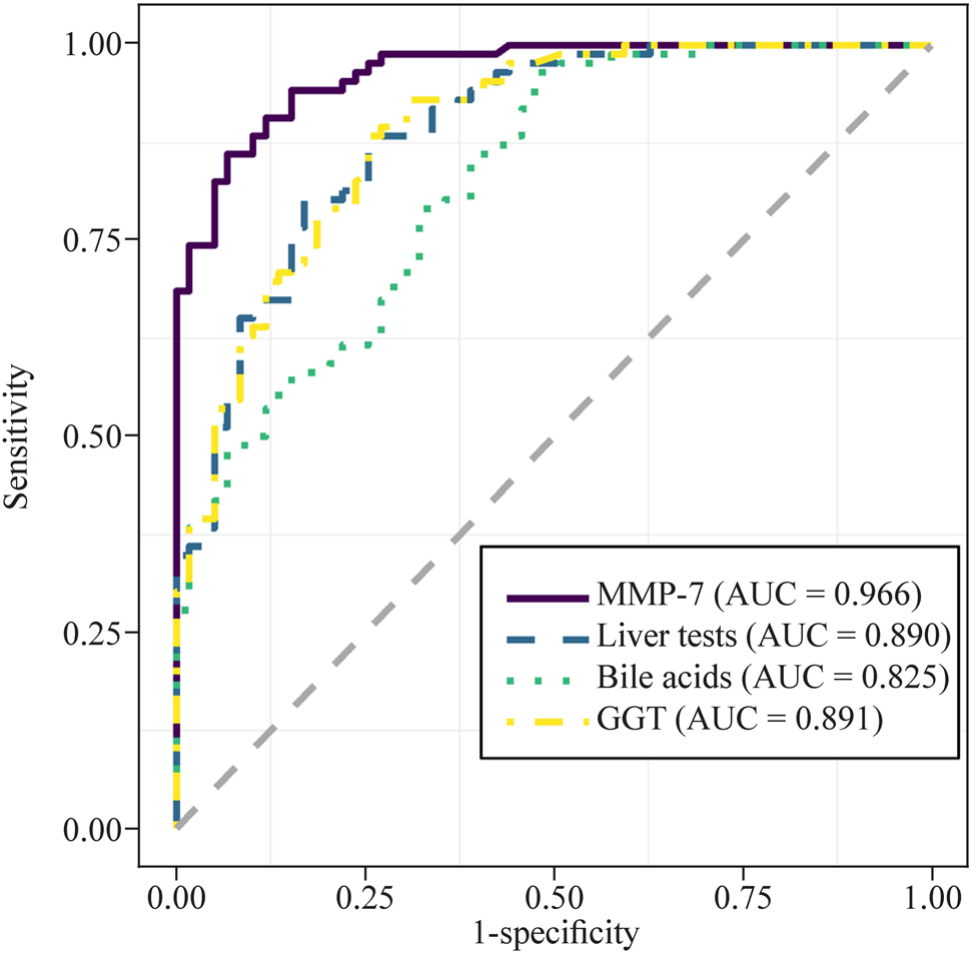
The prediction model was based on MMP-7 alone, liver test results (including GGT levels and the A/G ratio), bile acid levels [including TUDCA levels, the GUDCA to UDCA ratio, log_2_(GUDCA to UDCA ratio + 1)], and GGT. ROC plot showing the efficiency of the models for distinguishing BA and non-BA. (BA: *n* = 86, non-BA: *n* = 59). *MMP-7* Matrix metalloproteinase-7, *BA* biliary atresia, *GGT* gamma-glutamyl transferase, *A/G* albumin to globulin (A/G) ratio, *ROC* receiver operating characteristic, *AUC* area under the ROC curve, *UDCA* ursodeoxycholic acid, *GUDCA* glycoursodeoxycholic acid, *TUDCA* tauroursodeoxycholic acid

The model with the lowest AIC was chosen as the model that achieved the best tradeoff between fit and model complexity. Subsequently, a computational prediction model was constructed utilizing various variables, including MMP-7 expression levels, liver test results (including GGT levels and the A/G ratio), and bile acid levels [including TUDCA levels, the GUDCA to UDCA ratio, log_2_ (GUDCA to UDCA ratio + 1)] (Fig. 3). The results revealed that MMP-7 levels alone had the highest predictive ability, with an area under the curve (AUC) of 0.966 [95% confidence interval (CI): 0.942, 0.989], a sensitivity of 0.860, a specificity of 0.932, and an accuracy of 0.890. The AUC was 0.890 (95% CI 0.837, 0.943) for liver test results (including the A/G ratio and GGT levels), with a sensitivity of 0.802, a specificity of 0.831, and an accuracy of 0.814. The AUC was 0.825 (95% CI 0.758, 0.892) for bile acid levels [including TUDCA levels, the GUDCA to UDCA ratio, log_2_ (GUDCA to UDCA ratio + 1)], with a sensitivity of 0.965, a specificity of 0.525, and an accuracy of 0.786. Additionally, a computational prediction model based on GGT levels was constructed, with an AUC of 0.891 (95% CI 0.838, 0.943), a sensitivity of 0.884, a specificity of 0.746, and an accuracy of 0.828. Detailed comparison results for these indicators can be found in Supplementary Table 3. In this study, the cutoff value for using MMP-7 alone in diagnosing BA was 28.575 ng/mL.

### Predictive ability of MMP-7 levels combined with bile acid levels had better performance

**Fig. 4:**
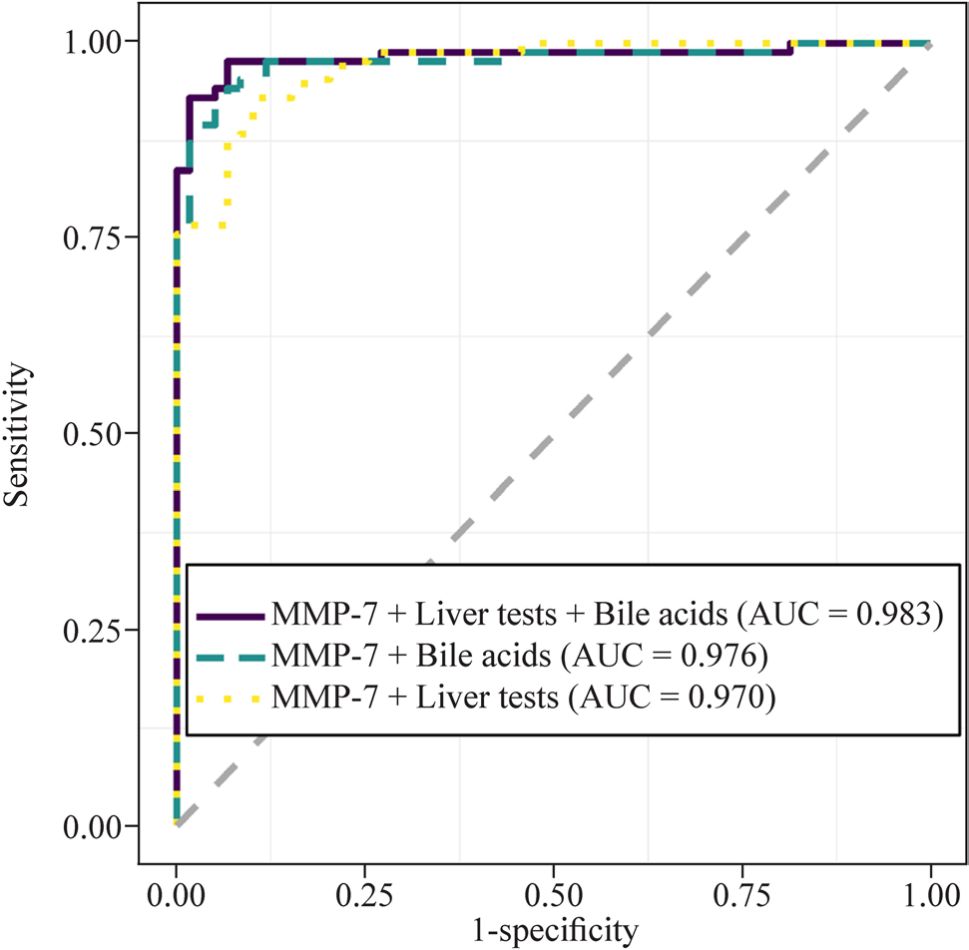
The prediction model based on MMP-7 combined with liver tests and bile acids. ROC plot showing the efficiency of the models for distinguishing BA and non-BA. (BA: *n* = 86, non-BA: *n* = 59). *MMP-7* matrix metalloproteinase-7, *BA* biliary atresia, *ROC* receiver operating characteristic, *AUC* area under the ROC curve

Integrated computational models were constructed based on the combination of MMP-7 expression levels and other indicators (Fig. 4). The results revealed that bile acid levels, including TUDCA levels and log_2_ (GUDCA to UDCA ratio + 1), provided a greater contribution to the predictive ability of MMP-7 levels for BA diagnosis, and the AUC reached 0.976 (95% CI 0.953, 1.000), with a sensitivity of 0.942, a specificity of 0.932, and an accuracy of 0.938. The AUC was 0.970 (95% CI 0.948, 0.992) for MMP-7 levels combined with liver test results, including the A/G ratio, with a sensitivity of 0.930, a specificity of 0.898, and an accuracy of 0.917. The AUC was 0.983 (95% CI 0.962, 1.000) for MMP-7 levels combined with liver test results and bile acid levels, including the A/G ratio, TUDCA levels, the GCDCA to CDCA ratio, and log_2_ (GCA to CA ratio + 1), with a sensitivity of 0.930, a specificity of 0.983, and an accuracy of 0.952. The detailed comparison results of these indicators are shown in Supplementary Table 3.

**Fig. 5:**
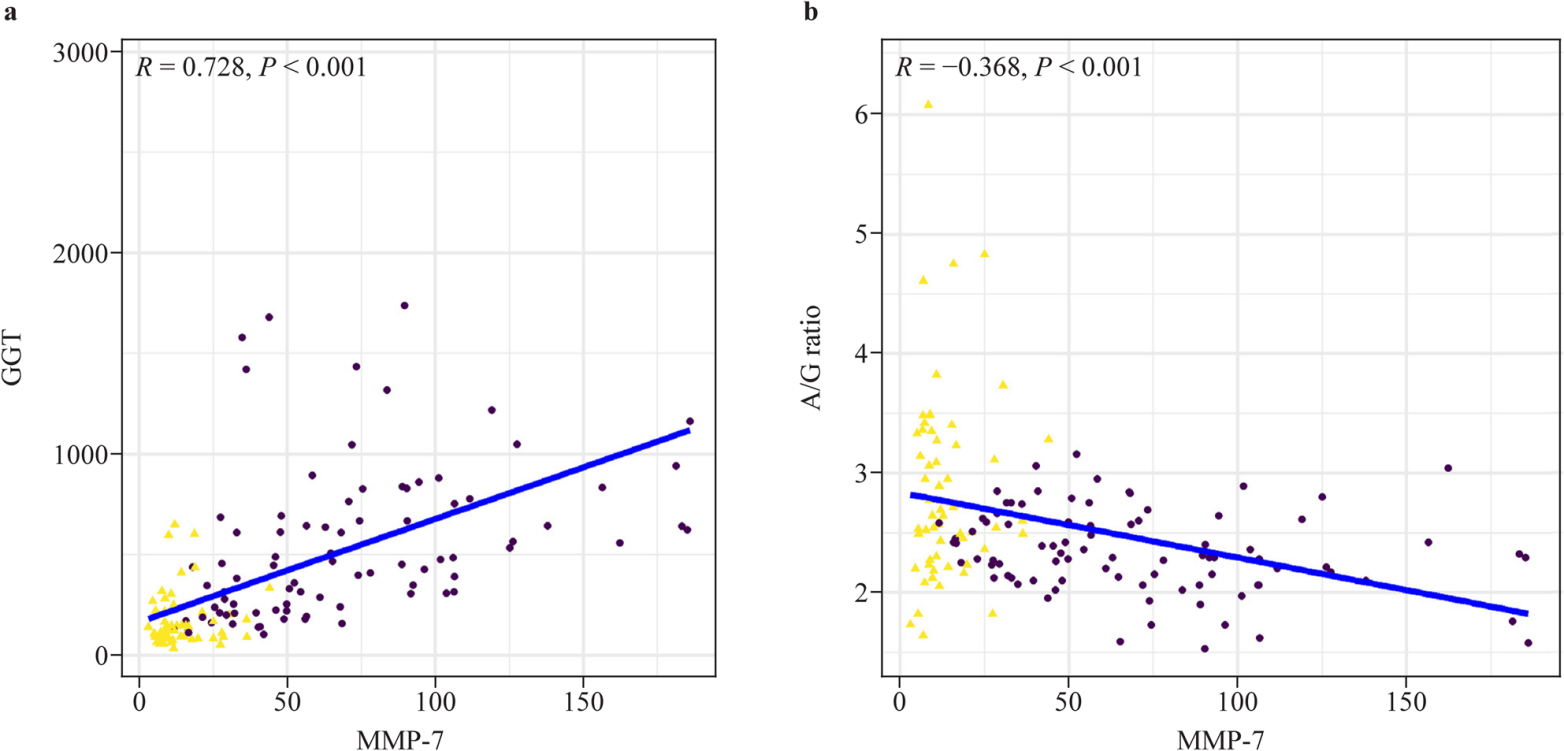
Correlation analysis of serum MMP-7 expression level with GGT and A/G ratio. **a** is the Spearman’s rank correlation coefficient between MMP-7 and GGT. **b** shows the Spearman’s rank correlation coefficient between MMP-7 and the A/G ratio. *MMP-7* Matrix metalloproteinase-7, *GGT* gamma-glutamyl transferase, *A/G* Albumin to globulin (A/G) ratio

In an effort to comprehend why GGT levels demonstrated a suboptimal role in aiding the diagnosis of BA with MMP-7 levels in contrast to the A/G ratio, the correlation between MMP-7 expression levels and GGT levels as well as the A/G ratio were calculated. The results indicated that MMP-7 levels had a significantly positive correlation with GGT levels (*P* < 0.0001), with a correlation coefficient of 0.728 (Fig. 5a). On the other hand, MMP-7 levels had a significantly negative correlation with the A/G ratio (*P* < 0.001), with a correlation coefficient of - 0.368 (Fig. 5b). We consequently posit that the diminished efficacy of GGT levels in facilitating the diagnosis of BA could be ascribed to its more pronounced positive correlation and therefore significant collinearity with MMP-7 expression levels.

### Liiver fibrosis score was significantly correlated with serum MMP-7 levels, specific liver test results, and levels of specific bile acids

**Fig. 6:**
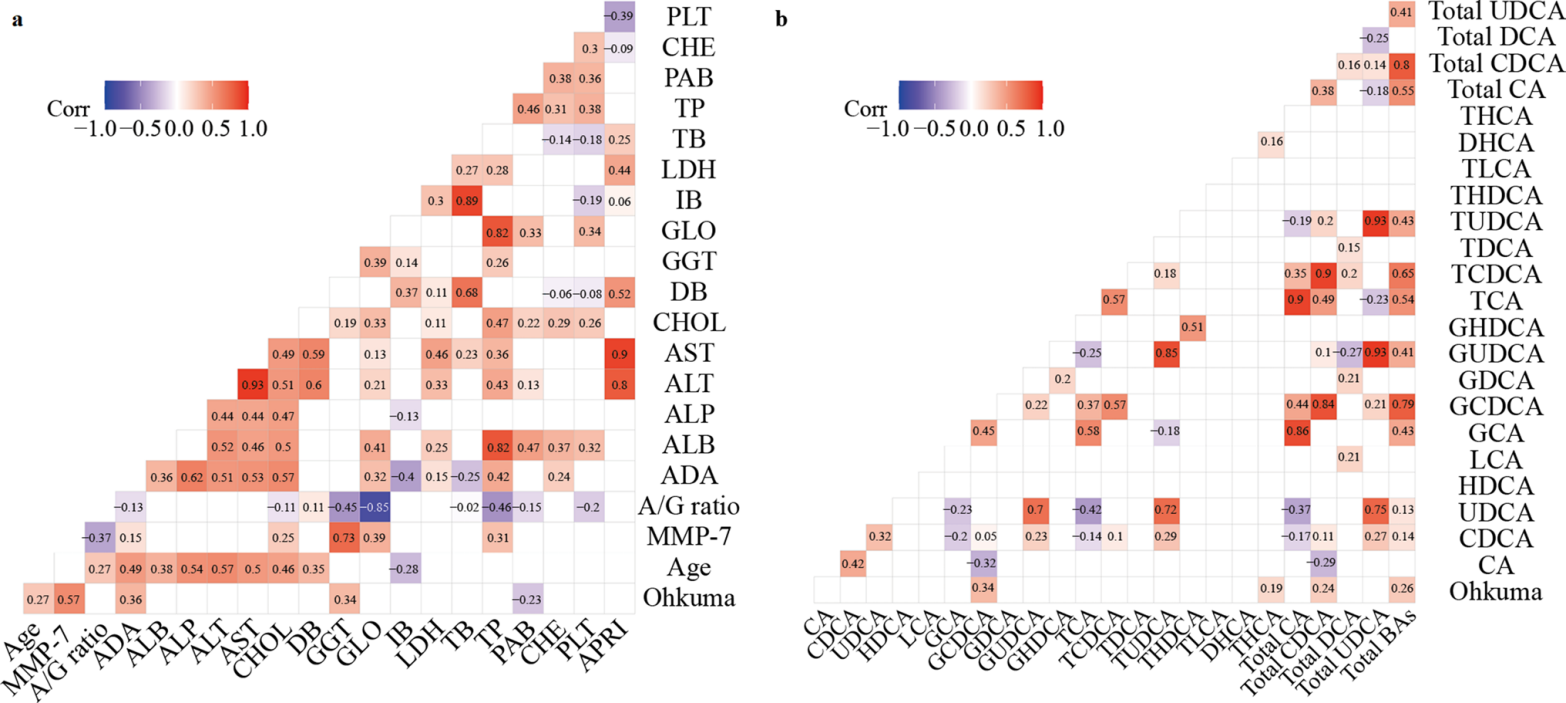
Correlation of liver fibrosis score with age, serum MMP-7, liver tests, and bile acids. **a** Spearman’s rank correlation coefficient among liver fibrosis score, age, MMP-7 expression level and liver tests. **b** was the Spearman’s rank correlation coefficient among liver fibrosis score and bile acids. A statistically significant difference was defined as a P value < 0.05, and we only showed the correlation coefficients of the significantly different comparisons*. MMP-7* matrix metalloproteinase-7, *A/G* albumin to globulin (A/G) ratio, *ADA* adenosine deaminase, *ALB* albumin, *ALP* alkaline phosphatase, *ALT* alanine transaminase, *AST* aspartate aminotransferase, *CHOL* cholesterol, *DB* direct bilirubin, *GGT* gamma-glutamyl transferase, *GLO* globulin, *IB* indirect bilirubin, *LDH* lactate dehydrogenase, *TB* total bilirubin, *TP* total protein, *PAB* prealbumin, *CHE* cholinesterase, *PLT* platelet, *APRI* aspartate aminotransferase to platelet ratio index, *CA* cholic acid, *CDCA* chenodeoxycholic acid, *UDCA* ursodeoxycholic acid, *HDCA* hyodeoxycholic acid, *LCA* lithocholic acid, *GCA* glycocholic acid, *GCDCA* glycochenodeoxycholic acid, *GDC*A glycodeoxycholic acid, *GUDCA* glycoursodeoxycholic acid, *GHDCA* glycohyodeoxycholic acid, *TCA* taurocholic acid, *TCDCA* taurochenodeoxycholic acid, *TDCA* taurodeoxychoic acid, *TUDCA* tauroursodeoxycholic acid, *THDCA* taurohyodeoxycholic acid, *TLCA* taurolithocholic acid, *DHCA* dihydroxycholestanoic acid, *THCA* trihydroxycholestanoic acid, *total CA* CA + TCA + GCA, *total CDCA* CDCA + TCDCA + GCDCA, *total DCA* DCA + TDCA + GDCA, *total UDCA* UDCA + TUDCA + GUDCA, *total BAs* Total CA + total CDCA + total DCA + total UDCA

The liver fibrosis score was determined using the Ohkuma classification method, which assigns grades of 0–4. Our results indicated a significant positive correlation between the liver fibrosis score and MMP-7 expression levels (*P* < 0.05), with a correlation coefficient of 0.57 (Fig. 6a). In addition, we observed significant positive correlations between the liver fibrosis score and ADA and GGT levels, while a significant negative correlation was found with PAB levels (Fig. 6a). Among the correlation analyses between the liver fibrosis score and bile acid levels, only GCDCA levels, THCA levels, Total CDCA, and Total BAs (bile acids) were significantly positively correlated with the liver fibrosis score (Fig. 6b). We also observed a significant positive correlation between the liver fibrosis score and age (Fig. 6a), and all the other significantly correlated results are shown in Fig. 6a & b.

## Discussion

BA is a rare and fatal liver disease that affects children, and early diagnosis is crucial [25, 26]. To this end, we conducted a study in which we determined the levels of MMP-7, liver test results, and bile acid levels in 86 BA patients and 59 non-BA patients. We then developed integrated computational models to diagnose BA based on these indicators. Our study revealed four major findings. First, we found significant differences in MMP-7 levels, the results of four liver tests, and the levels of ten bile acids between BA and non-BA patients. Second, we found that MMP-7 levels were highly correlated with GGT levels and the liver fibrosis score. Third, our computational prediction model demonstrated that MMP-7 levels alone had higher predictive accuracy than liver test results and bile acid levels. Fourth, bile acids contributed more significantly to enhancing the predictive accuracy of MMP-7 levels than liver test results. All the techniques employed in this study are clinically applicable. Our innovative integrated models, based on a large number of indicators, provide a noninvasive and cost-effective approach for accurately diagnosing BA.

In recent years, an increasing number of studies have reported that serum MMP-7 levels show promising diagnostic potential for future clinical application [12, 13]. Previous studies revealed that MMP-7 levels were significantly higher in BA patients than in non-BA patients [27, 28]; however, different studies often had different expression values. For example, Jiang J et al. found that the median serum MMP-7 levels were 38.39 ng/mL for the BA group and 4.4 ng/mL for the non-BA group [29]. In our study, we found that the median serum MMP-7 level for the BA patients was 61.95 ng/mL and that the median serum MMP-7 level for the non-BA patients was 10.79 ng/mL. It has been reported that MMP-7 expression levels may vary based on factors including ethnicity, patient age, and the experimental reagents used for MMP-7 measurement [12, 30].

Liver tests play a crucial role in the diagnosis of BA. Ranucci G et al. reported that bilirubin, ALT, AST, ALP, GGT, TP and ALB levels were used to define the severity of liver involvement [22]. Elevated levels of bilirubin, particularly DB, are hallmarks of BA [31]. Previous studies reported that DB levels determined using liver tests were elevated in all 34 BA patients at 24-72 hours [1]. Elevated GGT levels often accompany cholestasis, making it another valuable marker for BA diagnosis [32]. Increased ALP levels suggest cholestasis, a condition where bile flow is obstructed. While not specific to BA, elevated levels of AST and ALT can indicate liver inflammation [33]. In this study, we identified that four liver test indicators were significantly different, including GGT, TP, and GLO levels and the A/G ratio, between BA and non-BA patients. These significantly changed indicators will potentially contribute more to the diagnosis of BA.

Many studies have conducted further research on the predictive ability of MMP-7 levels for the diagnosis of BA [22, 34–36]. Wu et al. found that serum MMP-7 levels greater than 1.43 ng/mL could predict BA in infants with cholestasis, with an accuracy of 88%. When the serum MMP-7 level was greater than 1.43 ng/mL, the positive predictive value, negative predictive value, and diagnostic accuracy of BA were 76.07%, 98.15%, and 88%, respectively [12]. Yang L et al. detected serum MMP-7 levels in 135 infants under six months of age with cholestasis and established the optimal MMP-7 cutoff value in BA patients as 52.85 ng/mL, with a sensitivity of 98.67%, a specificity of 95.00%, and a negative predictive value of 98.28% [8]. Similarly, in the latest study, Singh TR et al. confirmed the diagnostic value of serum MMP-7 levels for BA through a prospective study and established the serum MMP-7 cutoff value as 4.99 ng/mL, with a sensitivity, specificity, and negative predictive value of 96%, 90.4%, and 95%, respectively [37]. It can be concluded from these studies that MMP-7 levels play an important role in the diagnosis of BA, but different studies have not reached a consensus on the cutoff value of MMP-7 for the diagnosis of BA [27]. In fact, most of these studies were not of Chinese origin. In this study, the cutoff value for using MMP-7 levels alone in diagnosing BA was 28.575 ng/mL, with an AUC of 0.966, a sensitivity of 0.860, a specificity of 0.932, and an accuracy of 0.890. The discrepancies in cutoff values among various studies can be attributed to several factors. First, variation in the laboratory techniques and assay methods used for MMP-7 measurement can lead to discrepancies. Different studies might employ distinct assay kits or platforms, which can yield varying results. Second, differences in the demographic and clinical characteristics of the study populations can significantly impact the choice of cutoff values. For instance, age, sex, and ethnicity can influence the levels of MMP-7. These results provide new accurate evidence for the use of MMP-7 levels, which hold greater reference value for the Chinese population.

Liver test results and bile acid levels were reported to be promising diagnostic tools for BA [17, 35, 38]. Ranucci et al. revealed that high GGT levels were the extrahepatic cause of BA [22]. Wu et al. reported that GGT levels had a higher AUC for BA diagnosis than MMP-7 levels [39]; however, most studies revealed that MMP-7 levels had a higher AUC for BA diagnosis than GGT levels [29, 30, 40]. These inconsistent results limit the usage of MMP-7 and GGT levels for clinical diagnosis; therefore, more reliable studies are needed. In the present study, the predicted AUC was 0.891 using GGT levels alone for the diagnosis of BA, which was lower than the AUC of MMP-7 levels. We found that the combination of GGT levels and the A/G ratio had higher specificity for BA diagnosis than GGT levels alone. Through selection according to the AIC, three bile acids [including TUDCA, the GUDCA to UDCA ratio, and log_2_ (GUDCA to UDCA ratio + 1)] were selected to predict BA diagnosis, and the AUC was also relatively high but lower than that of MMP-7 and GGT levels. These findings indicated that the combination of the results of two or three liver tests and bile acid levels could also be utilized for diagnosing BA, although the predictive ability was slightly lower than that of MMP-7 expression levels.

Integrated models allow for a comprehensive understanding of complex systems by incorporating multiple variables and their interactions [41, 42]. However, there is a lack of integrated models that combine MMP-7 levels with liver test results and bile acid levels for diagnosing BA. In this study, we constructed integrated computational models to diagnose BA based on the combination of MMP-7 levels, liver test results, and bile acid levels. We found that the integrated models had a higher predictive accuracy than the model with MMP-7 levels alone. Additionally, we discovered that bile acid levels contributed more to the predictive ability of MMP-7 levels for BA diagnosis than liver test results. These findings highlight the potential of bile acid levels and liver test results to enhance the accuracy of diagnosing BA using MMP-7 expression levels. This approach provides a fresh perspective and a more accurate and reliable method for diagnosing BA.

Lertudomphonwanit et al. reported that serum MMP-7 levels correlated poorly with liver fibrosis [13]. However, most studies have reported that MMP-7 levels are positively correlated with liver fibrosis [43–45]. In this study, we found that MMP-7 levels were highly correlated with liver fibrosis, which is consistent with most studies. In addition, we discovered that the results of some liver tests (including ADA, GGT and PAB levels) were significantly correlated with liver fibrosis, and the levels of some bile acids (including GCDCA, THCA, Total CDCA, and Total BAs) were significantly correlated with liver fibrosis. Therefore, our study identified more liver tests and bile acids that could be used to determine the degree of liver fibrosis.

This study has several limitations. First, although we included 86 BA patients and 59 non-BA patients, the sample size of this study was relatively small. Second, our study was conducted at a single medical center, which may limit the generalizability of our findings. Third, we established cutoff values for MMP-7 and other indicators, and these values may need further validation in larger and more diverse patient cohorts. The optimal cutoff values may vary in different populations. Fourth, our study was retrospective in nature, which may introduce selection bias. Apart from the limitations, this study has several strengths. First, this study employed a comprehensive approach by determining MMP-7 expression levels, liver test results, and bile acid levels in BA patients and non-BA patients. This multifaceted assessment allows for a more holistic understanding of BA diagnosis. Second, we developed innovative integrated computational models that combine MMP-7 levels with liver test results and bile acid levels. These integrated models demonstrated higher predictive accuracy than the model using MMP-7 levels alone, offering a valuable tool for accurate BA diagnosis. Third, all the techniques and assays used in our study are clinically applicable, making our findings relevant for potential clinical implementation. Fourth, our study not only focused on BA diagnosis but also explored the correlation among MMP-7 levels, liver test results, bile acid levels, and liver fibrosis. This additional information can aid in understanding the progression of the disease.

In conclusion, this study presents a comprehensive approach to the early diagnosis of BA, a rare and life-threatening liver disease in children. We demonstrated significant differences in serum MMP-7 levels, liver test results, and bile acid levels between BA and non-BA patients. MMP-7 levels, a promising biomarker for BA, showed remarkable diagnostic accuracy when used alone. Moreover, the integration of liver test results and bile acid levels with MMP-7 levels improved diagnostic accuracy, providing a more reliable noninvasive method for BA diagnosis. We also observed significant correlations among MMP-7 levels, liver fibrosis, the results of specific liver tests and bile acid levels, shedding light on their potential roles in assessing disease severity. These findings offer valuable insights into the development of accurate and timely diagnostic approaches for BA, ultimately enhancing patient outcomes and reducing the need for invasive procedures. These biomarkers can also reflect the extent of liver fibrosis in pediatric patients, potentially guiding physicians in determining the timing of liver transplantation.

## Supplementary Information

Below is the link to the electronic supplementary material.

Supplementary file1 (DOCX 80 KB)

## Data Availability

The data that support the findings of this study are available from the corresponding author upon reasonable request.

## References

[1] Bezerra JA, Wells RG, Mack CL, Karpen SJ, Hoofnagle JH, Doo E, et al. Biliary atresia: clinical and research challenges for the twenty-first century. Hepatology. 2018;68:1163–73. 29604222 10.1002/hep.29905 PMC6167205

[2] Mohanty SK, Donnelly B, Temple H, Ortiz-Perez A, Mowery S, Lobeck I, et al, Tiao G. High Mobility Group Box 1 Release by Cholangiocytes Governs Biliary Atresia Pathogenesis and Correlates With Increases in Afflicted Infants. Hepatology. 2021;74:864–78. 33559243 10.1002/hep.31745 PMC8349381

[3] Liu X, Peng X, Huang Y, Shu C, Liu P, Xie W, et al. Design and validation of a noninvasive diagnostic criteria for biliary atresia in infants based on the STROBE compliant. Medicine (Baltimore). 2019;98:e13837. 30732123 10.1097/MD.0000000000013837 PMC6380858

[4] Lai MW. Challenges in the diagnosis of biliary atresia in cholestatic neonates. Pediatr Neonatol. 2023;64:3–4. 36550017 10.1016/j.pedneo.2022.12.002

[5] Lendahl U, Lui VCH, Chung PHY, Tam PKH. Biliary atresia - emerging diagnostic and therapy opportunities. EBioMedicine. 2021;74:103689. 34781099 10.1016/j.ebiom.2021.103689 PMC8604670

[6] Lyu H, Ye Y, Lui VCH, Wu W, Chung PHY, Wong KKY, et al. Plasma amyloid-beta levels correlated with impaired hepatic functions: an adjuvant biomarker for the diagnosis of biliary atresia. Front Surg. 2022;9:931637. 36132201 10.3389/fsurg.2022.931637 PMC9483031

[7] Schreiber RA. Newborn screening for biliary atresia. JAMA. 2020;323:1137–8. 32207779 10.1001/jama.2020.2727

[8] Yang L, Zhou Y, Xu PP, Mourya R, Lei HY, Cao GQ, et al. Diagnostic accuracy of serum matrix metalloproteinase-7 for biliary atresia. Hepatology. 2018;68:2069–77. 30153340 10.1002/hep.30234 PMC6519383

[9] Chi S, Xu P, Yu P, Cao G, Wang H, Ye Y, et al. Dynamic analysis of serum MMP-7 and its relationship with disease progression in biliary atresia: a multicenter prospective study. Hepatol Int. 2022;16:954–63. 35729470 10.1007/s12072-022-10322-x

[10] Gunda ST, Chambara N, Chen XF, Pang MYC, Ying MT. Diagnostic efficacy of advanced ultrasonography imaging techniques in infants with biliary atresia (BA): a systematic review and meta-analysis. Children (Basel). 2022;9:1676. 36360404 10.3390/children9111676 PMC9688715

[11] Zhao J, Zhao Y, Zhang Y, Liao J, Li S, Wang D, et al. Preliminary exploration of the efficacy of laparoscopic fluorescence cholangiography (LFC) in the diagnosis of biliary atresia compared with intraoperative cholangiography (IOC). Photodiagn Photodyn Ther. 2023;41:103241. 10.1016/j.pdpdt.2022.103241 36528283

[12] Wu JF, Jeng YM, Chen HL, Ni YH, Hsu HY, Chang MH. Quantification of serum matrix metallopeptide 7 levels may assist in the diagnosis and predict the outcome for patients with biliary atresia. J Pediatr. 2019;208:30-7e1. 30853207 10.1016/j.jpeds.2018.12.006

[13] Lertudomphonwanit C, Mourya R, Fei L, Zhang Y, Gutta S, Yang L, et al. Large-scale proteomics identifies MMP-7 as a sentinel of epithelial injury and of biliary atresia. Sci Transl Med. 2017;9:eaan8462. 29167395 10.1126/scitranslmed.aan8462 PMC5902315

[14] Harpavat S. MMP-7: the next best serum biomarker for biliary atresia? J Pediatr. 2019;208:8–9. 30857775 10.1016/j.jpeds.2019.01.026

[15] Johansson H, Svensson JF, Almstrom M, Van Hul N, Rudling M, Angelin B, et al. Regulation of bile acid metabolism in biliary atresia: reduction of FGF19 by Kasai portoenterostomy and possible relation to early outcome. J Intern Med. 2020;287:534–45. 31976601 10.1111/joim.13028

[16] Farooqui N, Elhence A, Shalimar E. A current understanding of bile acids in chronic liver disease. J Clin Exp Hepatol. 2022;12:155–73. 35068796 10.1016/j.jceh.2021.08.017 PMC8766695

[17] Zhou K, Wang J, Xie G, Zhou Y, Yan W, Pan W, et al. Distinct plasma bile acid profiles of biliary atresia and neonatal hepatitis syndrome. J Proteome Res. 2015;14:4844–50. 26449593 10.1021/acs.jproteome.5b00676

[18] Rabbani T, Guthery SL, Himes R, Shneider BL, Harpavat S. Newborn screening for biliary atresia: a review of current methods. Curr Gastroenterol Rep. 2021;23:28. 34817690 10.1007/s11894-021-00825-2 PMC8651301

[19] Dong R, Jiang J, Zhang S, Shen Z, Chen G, Huang Y, et al. Development and validation of novel diagnostic models for biliary atresia in a large cohort of Chinese patients. EBioMedicine. 2018;34:223–30. 30077722 10.1016/j.ebiom.2018.07.025 PMC6116426

[20] Yan H, Liu J, Jin S, Du L, Wang Q, Luo Y. A novel prediction tool based on shear wave elastography, gallbladder ultrasound, and serum biomarkers for the early diagnosis of biliary atresia in infants younger than 60 days old. Quant Imaging Med Surg. 2023;13:259–70. 36620159 10.21037/qims-22-324 PMC9816742

[21] Zhao D, Zhou K, Chen Y, Xie W, Zhang Y. Development and validation of bile acid profile-based scoring system for identification of biliary atresia: a prospective study. BMC Pediatr. 2020;20:255. 32460787 10.1186/s12887-020-02169-8 PMC7251733

[22] Ranucci G, Della Corte C, Alberti D, Bondioni MP, Boroni G, Calvo PL, et al. Diagnostic approach to neonatal and infantile cholestasis: a position paper by the SIGENP liver disease working group. Dig Liver Dis. 2022;54:40–53. 34688573 10.1016/j.dld.2021.09.011

[23] Fumino S, Higuchi K, Aoi S, Furukawa T, Kimura O, Tajiri T. Clinical analysis of liver fibrosis in choledochal cyst. Pediatr Surg Int. 2013;29:1097–102. 23975015 10.1007/s00383-013-3368-7

[24] Lee MH, Shin HJ, Yoon H, Han SJ, Koh H, Lee MJ. Periportal thickening on magnetic resonance imaging for hepatic fibrosis in infantile cholestasis. World J Gastroenterol. 2020;26:2821–30. 32550757 10.3748/wjg.v26.i21.2821 PMC7284183

[25] Zhu JJ, Yang YF, Dong R, Zheng S. Biliatresone: progress in biliary atresia study. World J Pediatr. 2023;19:417–24. 36166189 10.1007/s12519-022-00619-0 PMC10149470

[26] Goodhue C, Fenlon M, Wang KS. Newborn screening for biliary atresia in the United States. Pediatr Surg Int. 2017;33:1315–8. 28983658 10.1007/s00383-017-4159-3

[27] Sakaguchi H, Konishi KI, Yasuda R, Sasaki H, Yoshimaru K, Tainaka T, et al. Serum matrix metalloproteinase-7 in biliary atresia: a Japanese multicenter study. Hepatol Res. 2022;52:479–87. 35106887 10.1111/hepr.13753

[28] He L, Ip DKM, Tam G, Lui VCH, Tam PKH, Chung PHY. Biomarkers for the diagnosis and post-Kasai portoenterostomy prognosis of biliary atresia: a systematic review and meta-analysis. Sci Rep. 2021;11:11692. 34083585 10.1038/s41598-021-91072-y PMC8175424

[29] Jiang J, Wang J, Shen Z, Lu X, Chen G, Huang Y, et al. Serum MMP-7 in the diagnosis of biliary atresia. Pediatrics. 2019;144:e20190902. 31604829 10.1542/peds.2019-0902

[30] Rohani P, Mirrahimi SB, Bashirirad H, Rahmani P, Kamran N, Alimadadi H, et al. Serum matrix metalloproteinase-7 levels in infants with cholestasis and biliary atresia. BMC Pediatr. 2022;22:351. 35717157 10.1186/s12887-022-03409-9 PMC9206322

[31] Harpavat S, Garcia-Prats JA, Anaya C, Brandt ML, Lupo PJ, Finegold MJ, et al. Diagnostic yield of newborn screening for biliary atresia using direct or conjugated bilirubin measurements. JAMA. 2020;323:1141–50. 32207797 10.1001/jama.2020.0837 PMC7093763

[32] Ihn K, Ho IG, Chang EY, Han SJ. Correlation between gamma-glutamyl transpeptidase activity and outcomes after Kasai portoenterostomy for biliary atresia. J Pediatr Surg. 2018;53:461–7. 29056230 10.1016/j.jpedsurg.2017.10.001

[33] Siu SL, Chan LW, Kwong AN. Clinical and biochemical characteristics of infants with prolonged neonatal jaundice. Hong Kong Med J. 2018;24:270–6. 29807952 10.12809/hkmj176990

[34] Lee CS, Ni YH, Chen HL, Wu JF, Hsu HY, Chien YH, et al. A pilot study of biliary atresia newborn screening using dried blood spot matrix metalloproteinase-7. J Pediatr Gastroenterol Nutr. 2023;76:418–23. 36946999 10.1097/MPG.0000000000003701

[35] Corrado MM, Mack CL. Diagnostic tools for early detection of biliary atresia: is a newborn screen attainable? Clin Liver Dis (Hoboken). 2022;19:25–8. 35106146 10.1002/cld.1165 PMC8785912

[36] Jiang J, Deng J, Chen G, Dong R, Sun S, Du M, et al. Protocol for a diagnostic accuracy study to develop diagnosis algorithm for biliary atresia using MMP-7 (DIABA-7 study): a study recruiting from Chinese Biliary Atresia Collaborative Network. BMJ Open. 2021;11:e052328. 10.1136/bmjopen-2021-052328

[37] Singh TR, Goel P, Bajpai M, Kandasamy D, Malik R, Yadav R, et al. Serum matrix metalloproteinase 7 as a diagnostic and prognostic biomarker for extrahepatic biliary atresia. J Indian Assoc Pediatr Surg. 2022;27:227–35. 35937114 10.4103/jiaps.JIAPS_389_20 PMC9350654

[38] Sun B, Kelleher S, Short C, Valencia PA, Zagory JA. Recent advancements in laboratory screening, diagnosis, and prognosis of biliary atresia: a literature review. Dig Med Res. 2021;4:1–10. 10.21037/dmr-21-52

[39] Wu B, Zhou Y, Tian X, Cai W, Xiao Y. Diagnostic values of plasma matrix metalloproteinase-7, interleukin-8, and gamma-glutamyl transferase in biliary atresia. Eur J Pediatr. 2022;181:3945–53. 36094664 10.1007/s00431-022-04612-7

[40] Tang X, Lv Y, Pu L, Ma J, Jin S, Xiang B. Matrix metalloproteinase-7 as a diagnostic marker for biliary atresia: a systematic review and meta-analysis. Indian J Surg. 2021;84:682–9. 10.1007/s12262-021-03107-3

[41] Jeon N, Lee H. Integrated fault diagnosis algorithm for motor sensors of in-wheel independent drive electric vehicles. Sensors (Basel). 2016;16:2106. 27973431 10.3390/s16122106 PMC5191086

[42] Pan F, Huang Y, Cai X, Wang Y, Guan Y, Deng J, et al. Integrated algorithm combining plasma biomarkers and cognitive assessments accurately predicts brain beta-amyloid pathology. Commun Med (Lond). 2023;3:65. 37165172 10.1038/s43856-023-00295-9 PMC10172320

[43] Geervliet E, Bansal R. Matrix metalloproteinases as potential biomarkers and therapeutic targets in liver diseases. Cells. 2020;9:1212. 32414178 10.3390/cells9051212 PMC7290342

[44] Kerola A, Lampela H, Lohi J, Heikkila P, Mutanen A, Hagstrom J, et al. Increased MMP-7 expression in biliary epithelium and serum underpins native liver fibrosis after successful portoenterostomy in biliary atresia. J Pathol Clin Res. 2016;2:187–98. 27499927 10.1002/cjp2.50 PMC4958739

[45] Wehrman A, Waisbourd-Zinman O, Wells RG. Recent advances in understanding biliary atresia. F1000Res. 2019;8:218. 10.12688/f1000research.16732.1 PMC6392153 30828434

