# Supplementary material for: Accurate prediction of biliary atresia with an integrated model using MMP-7 levels and bile acids
Source: World J Pediatr. 2023 Dec 23;20(8):822–33. doi: 10.1007/s12519-023-00779-7 (PMC11402860; doi:10.1007/s12519-023-00779-7)
Supplement: Supplementary file 2 — Supplementary file1 (DOCX 80 KB) [file 12519_2023_779_MOESM2_ESM.docx]

**Supplementary Fig. 1. Significantly different indicators of MMP-7 between BA and non-BA patients whose age were under 30 days and under 10 days.** The BA group comprised 27 patients, and the non-BA group comprised 6 patients. Yellow dots showed patients whose age were under 10 days. Among the subgroup, the BA group comprised 6 patients, and the non-BA group comprised 1 patient. Mann-Whitney U test was used for between-group comparisons, and a *P* value of less than 0.05 was considered statistically significant. *MMP-7* Matrix metalloproteinase-7, *BA* biliary atresia.

**
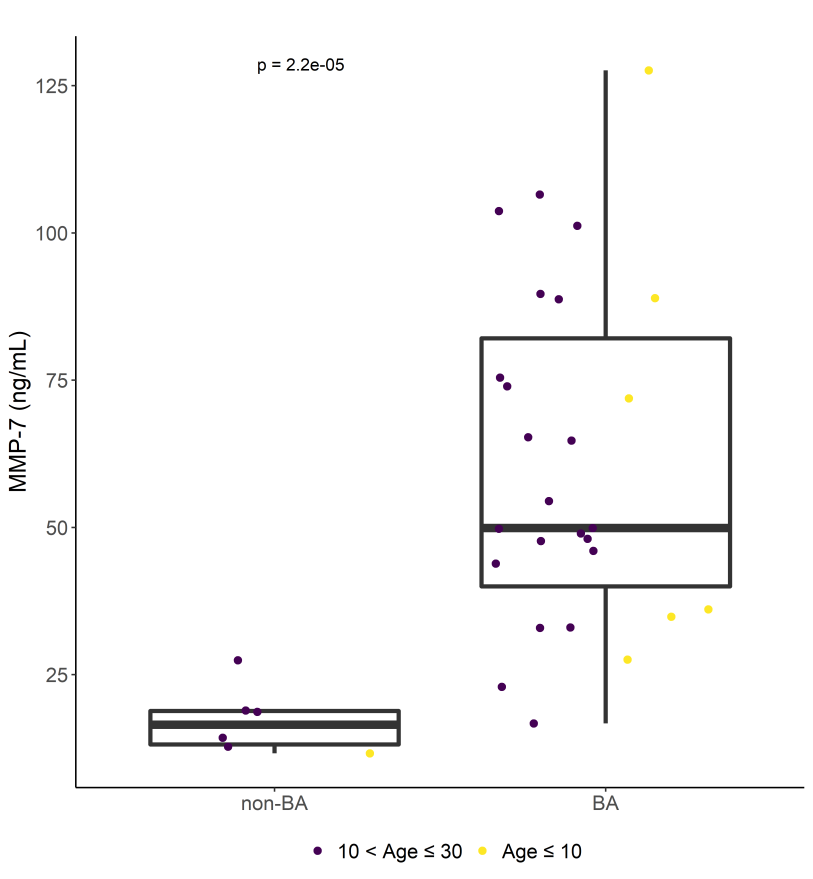
**

**Supplementary Table 1. Summary information of MMP-7 and liver test results of all the patients.**

| Diagnosis Status | Overall, N = 145 | BA, N = 86 | non-BA, N = 59 | p-value^a^ |
| --- | --- | --- | --- | --- |
| MMP-7 (ng/mL) | 31.91 (12.01, 70.75) | 61.95 (36.94, 92.94) | 10.79 (8.02, 16.25) | <0.001 |
| ALT (U/L) | 89.0 (41.0, 161.0) | 84.5 (38.2, 158.0) | 104.0 (43.0, 159.0) | 0.6 |
| AST (U/L) | 143.0 (73.0, 226.0) | 140.5 (72.2, 233.2) | 154.0 (77.5, 218.0) | 0.7 |
| ALP (U/L) | 541.0 (366.0, 694.0) | 547.0 (363.0, 700.8) | 503.0 (369.5, 666.0) | 0.5 |
| GGT (U/L) | 254.0 (129.0, 595.0) | 452.5 (243.5, 737.0) | 113.0 (85.0, 188.0) | <0.001 |
| TP (g/L) | 52.8 (48.9, 55.7) | 53.5 (50.0, 56.1) | 49.7 (45.0, 55.0) | 0.002 |
| ALB (g/L) | 37.0 (34.5, 39.4) | 37.2 (34.9, 39.1) | 36.7 (33.5, 39.6) | 0.3 |
| GLO (g/L) | 15.4 (13.2, 17.4) | 16.1 (14.3, 17.6) | 13.3 (10.9, 16.3) | <0.001 |
| A/G | 2.4 (2.2, 2.8) | 2.3 (2.1, 2.6) | 2.6 (2.3, 3.3) | <0.001 |
| TB (μmol/L) | 151.5 (117.8, 188.3) | 155.2 (124.3, 188.2) | 148.8 (108.8, 186.4) | 0.4 |
| DB (μmol/L) | 71.6 (52.0, 89.1) | 72.5 (52.6, 88.8) | 67.7 (50.8, 93.8) | >0.9 |
| IB (μmol/L) | 75.7 (58.0, 101.1) | 77.2 (62.6, 101.6) | 70.1 (47.2, 99.0) | 0.14 |
| ADA (U/L) | 8.4 (4.3, 15.7) | 10.4 (4.3, 16.7) | 6.9 (3.7, 13.8) | 0.079 |
| CHOL (mmol/L) | 3.6 (2.9, 4.8) | 3.7 (3.0, 4.8) | 3.4 (2.8, 4.5) | 0.092 |
| LDH (U/L) | 317.0 (266.0, 441.0) | 320.5 (261.5, 425.2) | 310.0 (269.5, 465.5) | 0.8 |
| PAB (mg/L) | 119.8 (100.7, 138.9) | 119.4 (103.8, 135.4) | 124.1 (94.2, 146.5) | >0.9 |
| CHE (U/L) | 6,081.0 (5,225.0, 7,101.0) | 6,211.5 (5,411.5, 7,023.5) | 5,796.0 (5,002.5, 7,129.0) | 0.2 |
| PLT (10^9^/L) | 408.0 (296.0, 495.0) | 420.0 (324.0, 495.0) | 371.0 (259.5, 482.5) | 0.056 |
| APRI | 0.6 (0.3, 1.2) | 0.5 (0.3, 1.0) | 0.8 (0.3, 1.5) | 0.2 |
| continuous variables are shown in median (IQR) | | | | |
| ^a^ Wilcoxon rank sum test | | | | |

*MMP-7* Matrix metalloproteinase-7, *BA* biliary atresia, *ALT* alanine transaminase, *AST* aspartate aminotransferase, *ALP* alkaline phosphatase, *GGT* gamma-glutamyl transferase, *TP* total protein, *ALB* albumin, *GLO* globulin, *A/G* albumin to globulin (A/G) ratio, *TB* total bilirubin, *DB* direct bilirubin, *IB* indirect bilirubin, *ADA* adenosine deaminase, *CHOL* cholesterol, *LDH* lactate dehydrogenase, *PAB* prealbumin, *CHE* cholinesterase, *PLT* platelet, *APRI* aspartate aminotransferase to platelet ratio index.

**Supplementary Table 2. The summary information of bile acids of all the patients.**

| Diagnosis Status | Overall, N = 145 | BA, N = 86 | non-BA, N = 59 | p-value^a^ |
| --- | --- | --- | --- | --- |
| CA | 0.000 (0.000, 0.000) | 0.000 (0.000, 0.000) | 0.000 (0.000, 0.000) | 0.038 |
| CDCA | 0.000 (0.000, 0.029) | 0.000 (0.000, 0.028) | 0.000 (0.000, 0.049) | 0.081 |
| DCA | 0.000 (0.000, 0.000) | 0.000 (0.000, 0.000) | 0.000 (0.000, 0.000) | 1.0 |
| UDCA | 0.000 (0.000, 0.417) | 0.000 (0.000, 0.000) | 0.278 (0.000, 1.832) | <0.001 |
| HDCA | 0.000 (0.000, 0.000) | 0.000 (0.000, 0.000) | 0.000 (0.000, 0.000) | 0.2 |
| LCA | 0.000 (0.000, 0.000) | 0.000 (0.000, 0.000) | 0.000 (0.000, 0.000) | 0.8 |
| GCA | 13.443 (6.736, 19.911) | 14.653 (10.264, 21.350) | 8.146 (3.664, 17.367) | <0.001 |
| GCDCA | 19.401 (11.515, 27.283) | 21.631 (15.527, 30.752) | 16.433 (9.061, 23.568) | 0.008 |
| GDCA | 0.000 (0.000, 0.000) | 0.000 (0.000, 0.000) | 0.000 (0.000, 0.000) | 0.8 |
| GUDCA | 0.280 (0.000, 13.698) | 0.000 (0.000, 2.769) | 6.435 (0.414, 20.581) | <0.001 |
| GHDCA | 0.000 (0.000, 0.000) | 0.000 (0.000, 0.000) | 0.000 (0.000, 0.000) | 0.4 |
| GLCA | 0.000 (0.000, 0.000) | 0.000 (0.000, 0.000) | 0.000 (0.000, 0.000) | 1.0 |
| TCA | 22.602 (12.158, 31.288) | 25.906 (19.294, 32.745) | 16.467 (9.270, 25.174) | 0.001 |
| TCDCA | 25.874 (15.826, 38.507) | 27.068 (19.073, 36.990) | 23.324 (13.145, 39.204) | 0.4 |
| TDCA | 0.000 (0.000, 0.000) | 0.000 (0.000, 0.000) | 0.000 (0.000, 0.000) | 0.4 |
| TUDCA | 0.093 (0.026, 6.635) | 0.036 (0.000, 0.907) | 6.546 (0.200, 16.548) | <0.001 |
| THDCA | 0.000 (0.000, 0.000) | 0.000 (0.000, 0.000) | 0.000 (0.000, 0.000) | 0.8 |
| TLCA | 0.000 (0.000, 0.000) | 0.000 (0.000, 0.000) | 0.000 (0.000, 0.000) | 0.2 |
| DHCA | 0.000 (0.000, 0.000) | 0.000 (0.000, 0.000) | 0.000 (0.000, 0.000) | 0.4 |
| THCA | 0.000 (0.000, 0.000) | 0.000 (0.000, 0.000) | 0.000 (0.000, 0.000) | 0.5 |
| Total_CA | 35.812 (23.869, 50.370) | 41.864 (29.015, 53.650) | 29.898 (14.244, 43.742) | 0.001 |
| Total_CDCA | 48.987 (30.617, 62.560) | 52.374 (38.035, 64.968) | 42.725 (25.366, 60.727) | 0.13 |
| Total_DCA | 0.002 (0.000, 0.013) | 0.005 (0.000, 0.015) | 0.000 (0.000, 0.006) | 0.003 |
| Total_UDCA | 0.359 (0.042, 25.139) | 0.061 (0.034, 5.763) | 21.960 (1.893, 43.290) | <0.001 |
| Total_BA | 101.909 (76.034, 124.887) | 102.195 (78.435, 127.616) | 101.170 (73.032, 123.560) | 0.8 |
| continuous variables are shown in median (IQR) | | | | |
| ^a^ Wilcoxon rank sum test | | | | |

*BA* biliary atresia, *CA* cholic acid, *CDCA* chenodeoxycholic acid, *DCA* deoxycholic acid, *UDCA* ursodeoxycholic acid, *HDCA* hyodeoxycholic acid, *LCA* lithocholic acid, *GCA* glycocholic acid, *GCDCA* glycochenodeoxycholic acid, *GDCA* glycodeoxycholic acid, *GUDCA* glycoursodeoxycholic acid, *GHDCA* glycohyodeoxycholic acid, *GLCA* glycolithocholic acid, *TCA* taurocholic acid, *TCDCA* taurochenodeoxycholic acid, *TDCA* taurodeoxychoic acid, *TUDCA* tauroursodeoxycholic acid, *THDCA* taurohyodeoxycholic acid, *TLCA* taurolithocholic acid, *DHCA* dihydroxycholestanoic acid, *THCA* trihydroxycholestanoic acid, *Total_CA* CA + TCA + GCA, *Total_CDCA* CDCA +TCDCA + GCDCA, *Total_DCA* DCA + TDCA + GDCA, *Total_UDCA* UDCA + TUDCA + GUDCA, *Total_BA* Total CA + Total CDCA + Total DCA + Total UDCA.

**Supplementary Table 3. Prediction model performances for different model inputs.**

| Model input | AUC (95% CI) | Sensitivity  (%) | Specificity  (%) | PPV  (%) | NPV  (%) | Accuracy  (%) |
| --- | --- | --- | --- | --- | --- | --- |
| MMP-7 + Liver tests + Bile acids | 0.983 (0.962, 1.000) | 93.0 | 98.3 | 98.8 | 90.6 | 95.2 |
| MMP-7 + Bile acids | 0.976 (0.953, 1.000) | 94.2 | 93.2 | 95.3 | 91.7 | 93.8 |
| MMP-7 + Liver tests | 0.970 (0.948, 0.992) | 93.0 | 89.8 | 93.0 | 89.8 | 91.7 |
| Liver tests | 0.890 (0.837, 0.943) | 80.2 | 83.1 | 87.3 | 74.2 | 81.4 |
| Bile acids | 0.825 (0.758, 0.892) | 96.5 | 52.5 | 74.8 | 91.2 | 78.6 |
| MMP-7 | 0.966 (0.942, 0.989) | 86.0 | 93.2 | 94.9 | 82.1 | 89.0 |
| GGT | 0.891 (0.838, 0.943) | 88.4 | 74.6 | 83.5 | 81.5 | 82.8 |

*MMP-7* Matrix metalloproteinase-7, *GGT* gamma-glutamyl transferase, *ROC* receiver operating characteristic, *AUC* area under the ROC curve, *PPV* positive predictive value, *NPV* negative predictive value.
